# Self-Learning about Herbal and Dietary Supplements: Perspectives Using the Theoretical Domains Framework

**DOI:** 10.3390/ijerph191710901

**Published:** 2022-09-01

**Authors:** Mohd Shahezwan Abd Wahab, Wan Nur Syamimi Wan Ismail, Aida Azlina Ali, Norkasihan Ibrahim, Noordin Othman, Nurul Aqmar Mohd Nor Hazalin, Khang Wen Goh, Long Chiau Ming

**Affiliations:** 1 Faculty of Pharmacy, Universiti Teknologi MARA (UiTM) Cawangan Selangor, Kampus Puncak Alam, Puncak Alam 42300, Malaysia; 2 Non-Destructive Biomedical and Pharmaceutical Research Centre, Smart Manufacturing Research Institute, Universiti Teknologi MARA (UiTM) Cawangan Selangor, Kampus Puncak Alam, Puncak Alam 42300, Malaysia; 3 Department of Clinical and Hospital Pharmacy, College of Pharmacy, Taibah University, Al-Madinah Al-Munawwarah 30001, Saudi Arabia; 4 School of Pharmacy, Management and Science University, Shah Alam 40100, Malaysia; 5 Faculty of Data Science and Information Technology, INTI International University, Nilai 71800, Malaysia; 6 PAP Rashidah Sa’adatul Bolkiah Institute of Health Sciences, Universiti Brunei Darussalam, Gadong BE1410, Brunei

**Keywords:** herbal and dietary supplements, focus group interview, pharmacy education, self-learning, theoretical domains framework

## Abstract

**Background:** Self-learning (SL) is a process in which individuals take the initiative to acquire knowledge with or without the help of others. Knowledge about herbal and dietary supplements (HDS) is important for pharmacists. Unfortunately, there is limited coverage of topics relating to HDS in the pharmacy curricula. The present focus group study applies the Theoretical Domains Framework (TDF) to explore pharmacy students’ practices and beliefs regarding SL about HDS (SL-HDS). **Methods:** Focus group interviews (FGIs) were conducted between April and May 2019 among a sample of undergraduate pharmacy students at a public university (*n* = 20). Four FGI sessions were conducted, each lasting about 60 to 75 min, and all the sessions were audio-recorded. The interviews were transcribed verbatim and analysed using thematic content analysis. **Results:** Beliefs about SL-HDS were categorised into 12 domains based on the TDF. Students showed positive attitudes towards SL-HDS and agreed that their involvement in SL-HDS was instrumental in improving their knowledge about various aspects of HDS including indications, adverse effects, and HDS-drug interactions. Various facilitators and barriers influencing students’ participation in SL-HDS were uncovered (e.g., access to the internet, time, availability of reference resources). The students demanded to be equipped with critical appraisal skills, as they had limited confidence in assessing literature or information about HDS. **Conclusion:** This study revealed that the students saw the benefits of SL-HDS. They also perceived that engaging in SL-HDS is compatible with the role of pharmacy students. The findings showed students’ readiness and willingness to conduct SL-HDS.

## 1. Introduction

Herbal and dietary supplements (HDS) are part of complementary and alternative medicine (CAM). There has been a global increase in the use of HDS by the public in recent years [1,2,3]. A Malaysian-based survey reported that the use of HDS is common among the public [4]. In the study, 17.1% and 29.6% of respondents were found to consume herbal supplements (HS) for the treatment of diseases and for health maintenance, respectively [4]. In the Malaysian Adults Nutrition Survey conducted in 2014, a higher percentage, namely 34% of respondents, were reported to consume HDS [5].

The use of HDS is a common practice not only among healthy individuals but also among chronically ill patients. For instance, a study reported that 44.5% of Malaysian patients consumed dietary supplements (DS), whereas 24.9% used HS to manage medical conditions including diabetes mellitus, dyslipidaemia, and peripheral neuropathy [6]. Furthermore, the use of HDS is prevalent among the elderly [7,8], pregnant women [9], and breastfeeding mothers [10]. As reported in previous studies, a growing interest and positive attitude towards HDS are two main reasons for the widespread use of HDS among the public and patients.

HDS products are readily accessible over the counter with no prescription required to obtain them. Thus, consumers can have the autonomy to use HDS for self-care. It has been demonstrated that the use of HDS is motivated by consumers’ proactive behaviour in taking care of their health [3,11], recommendations by their family or friends [12,13], and the influences of the media, especially through advertisements [14]. Nevertheless, it is important to note that information about HDS obtained from family and friends may be anecdotal, and information found in advertisements may include dubious promises and unproven claims [15]. This may potentially lead to inappropriate or irrational HDS use. 

Pharmacists are expected to encounter HDS frequently at all levels of care due to the accessibility and availability of HDS in retail pharmacies [16,17], and the widespread use of these products among patients. Pharmacists play an important role in educating consumers to ensure appropriate and safe use of HDS [18], so it is pertinent for them to be equipped with adequate knowledge regarding HDS. Nonetheless, previous studies have reported that many pharmacists perceived their knowledge of HDS as inadequate, thus affecting their confidence in discussing these products with consumers or patients [19]. This might have resulted from the limited content of HDS topics in pharmacy curricula [20]. 

In the United States, there is a growing interest among pharmacy schools in incorporating CAM content in their curricula. A survey in 2002 showed that only 73% of 63 pharmacy schools were offering CAM education [21]. Another study in 2003 showed that 80% of 64 pharmacy schools surveyed offered CAM and/or education on natural products [22]. A more recent survey among pharmacy schools in 2015 reported that all 96 pharmacy schools that responded to the survey incorporated CAM in their elective (*n* = 74) and required courses (*n* = 116) [23]. Of all the courses that incorporated CAM teachings (*n* = 190), 61.1% were required courses, whereas 38.9% were offered as electives. The study reported that 81.6% and 78.4% of the courses covered DS and HS topics, respectively. However, it should be noted that, although 116 required courses incorporated CAM in the content, only 14.7% of these courses focused exclusively on CAM, while the rest included CAM topics within the main content [23].

To date, there is a paucity of data on the extent of CAM education in Malaysian pharmacy curricula. However, reports from surveys conducted among Malaysian pharmacy students suggest that the content of CAM education in the local curricula is inadequate, and many students perceive their knowledge of CAM as insufficient [24,25,26]. Previous reports also showed that Malaysian pharmacy students were willing and ready to learn more about CAM and HDS during their studies [25,27,28].

Due to the rise in HDS use among the public and patients in Malaysia, it is imperative that Malaysian pharmacy graduates are knowledgeable about HDS. This, together with the positive acceptance among Malaysian pharmacy students of education in HDS, warrants pharmacy schools in Malaysia to put effort into ensuring that their graduates are equipped with HDS knowledge. That being said, introducing and incorporating HDS topics as a required or elective course in pharmacy education is difficult, due to the already condensed curricula that focus on the main topics of pharmacy education, e.g., pharmacology, clinical pharmacy, and pharmaceutics. 

One potential course of action to equip pharmacy students with HDS knowledge is through the inculcation of self-learning about HDS (SL-HDS) among them. Self-learning (SL) is, in fact, an essential skill that should be embraced by pharmacy students to ensure they become independent and life-long learners [29]. SL can be referred to as “a process in which individuals take the initiative, with or without the help of others, in diagnosing their learning needs, formulating goals, identifying human and material resources for learning, choosing and implementing appropriate learning strategies, and evaluating learning outcomes” [30]. In SL, it is the students rather than the teachers who determine the goals, learning methods, and resources [31]. Self-learners fill in the gaps of their formal education through discovery and reflection [32]. 

However, at present, there is no study that has been conducted to explore the practices of SL-HDS among pharmacy students. The beliefs about the factors (i.e., barriers and facilitators) associated with SL-HDS among pharmacy students are presently unknown. Therefore, the present qualitative study aims to employ the Theoretical Domains Framework (TDF) in exploring pharmacy students’ beliefs around the barriers to and facilitators of SL-HDS [33]. The TDF consists of 12 key theoretical domains related to the implementation of a behaviour: knowledge, skills, social/professional role and identity, beliefs about capabilities, beliefs about consequences, motivation and goals, memory/attention and decision processes, environmental context and resources, social influences (norms), emotion, behavioural regulation, and nature of the behaviour. 

The TDF has been employed to explore various behaviours in clinical settings, including the uptake of clinical guidelines among general practitioners [34], adverse drug reporting among nurses and pharmacists [35], and the engagement of physical activity among patients [36]. The use of the TDF in the context of the present study allows the elicitation of comprehensive factors that are associated with the performance of SL-HDS among pharmacy students. Findings from this study may provide insight about SL as a mode of learning about HDS among pharmacy students and may guide pharmacy educators in incorporating or promoting SL-HDS for their students. 

## 2. Methods

### 2.1. Study Design

This was a descriptive qualitative study using the focus group interview (FGI) among the first to fourth-year undergraduate pharmacy students at Universiti Teknologi MARA (UiTM), Malaysia. In this study, the FGI was chosen as the study design due to limited information regarding SL-HDS practices among pharmacy students. The FGI allows a detailed probing of the practices, thus providing robust data about the topic [37]. This study was approved by the Research Ethics Committee of UiTM, Malaysia (REC/235/19).

### 2.2. Study Informant Recruitment

The announcement of this study was advertised on the major social media platforms including WhatsApp, Facebook, and Instagram, and interested participants were required to contact one of the research team members (WNSWI). Study informants were purposively selected from the list of interested individuals to represent the first- to fourth-year students. The maximal variation sampling method was utilised to include students with a variation in cumulative grade point average (CGPA), gender, history of HDS use, and history of enrolment in the elective traditional medicine course. The target was to recruit five students from each study year. Students were eligible for participation in the FGI if they were undergraduate pharmacy students at UiTM, able to speak in the Malay language, and able to participate in the FGI at the time and date set by the researchers. A total of 20 students were selected and contacted by phone to arrange for the FGI. None of the contacted students refused to participate in the FGI. 

### 2.3. Discussion Question

The discussion questions (Table 1) were developed based on the TDF described earlier [33]. Five academic pharmacists who are experienced in pharmacy education research reviewed the discussion questions to examine the face validity. This was carried out to ensure that the discussion questions were suitable and relevant. Additionally, a pilot study was conducted on two undergraduate pharmacy students who were not involved in the main study. The pilot study showed that the discussion questions were easily understood and were adequate for an in-depth understanding of SL-HDS among informants. The participants’ information (demographic details) was collected using a demographic survey form. The discussion questions were translated into the local official language (i.e., Malay language), as recommended by a TDF guideline, and translated back to English for reporting purposes [37]. The final translation was checked and compared with the original version of the questions for accuracy of meaning and content. 

### 2.4. Data Collection

Four FGIs (one for each study year) were conducted between April and May 2019. Each FGI session was conducted by a trained pharmacy student (WNSWI) to maintain consistency in the interview method and approach. Each FGI session was conducted in the Malay language in small classrooms and lasted about 60 to 75 min. All FGIs were audio-recorded and field notes were taken by the interviewer throughout the process. The informants were assured of confidentiality and anonymity. Data saturation was reached when the data collected did not contribute to any new information about SL-HDS practices among the informants and the barriers and facilitators.

### 2.5. Data Analysis

The recording of the FGIs was transcribed verbatim by WNSWI and checked by MSAW. Anonymity was maintained by removing the names of students from the transcripts. A coding guide was developed to ensure consistent coding of the data collected from the informants. Two researchers (MSAW and AAA) coded the transcripts in Microsoft Word and Excel according to the 12 domains of TDF [38]. Furthermore, thematic content analysis was conducted independently [33], and both inductive and deductive approaches were employed in data analysis to prevent missing any theme. A good inter-rater reliability result (79.8%) was obtained between the two coders (MSAW and AAA) [37]. The two coders convened to discuss discrepancies and agreed on the final coding. The FGI and analysis were conducted in the Malay language. For the purpose of reporting, the quotes used to illustrate the identified themes were translated from Malay into English. The chosen quotes were presented to capture the meaning of the statements provided by the students, rather than a literal translation.

## 3. Results

### 3.1. Demographic Characteristics of Study Informants

A total of 20 students participated in the FGI with five students in each study year (Table 2). The mean age of the participants was 22.6 years old. The majority of the FGI participants were female (80%, 16/20). The majority of the students (75%, 15/20) had a CGPA ranging from 3.0 to 4.0, whereas 25% (5/20) of them had a CGPA of less than 3.0. As of the time of this study, six students (30%) were using at least one type of HDS. Of the 20 participants, seven (35%) had enrolled in the traditional medicine elective course.

### 3.2. Beliefs about SL-HDS

The frequency of specific beliefs regarding SL-HDS and the pattern of appearance of theoretical domains across the FGI sessions are presented in Table 3. The data were deemed to have reached the saturation point as no new theme was identified in the fourth FGI. 

#### 3.2.1. Knowledge about SL-HDS

Across study years, SL-HDS was admitted as an appropriate method to acquire more knowledge about HDS. Many students understood SL-HDS as informal learning and they mostly acquired knowledge about HDS from the internet. The students stated that SL-HDS could be conducted by performing their own research on HDS to enhance their understanding of the topic. For some students, HDS was perceived to be inadequately covered in the curriculum, so they conducted SL-HDS to supplement learning from their formal classes. The students acknowledged SL-HDS as a learning method without supervision. 

#### 3.2.2. Skills

The students were concerned about the level of skills required for them to conduct SL-HDS. Overall, the students agreed that, for them to conduct effective SL-HDS, they needed a strong foundation in pharmacy-related knowledge such as pharmacology and toxicology. The students affirmed that there is an abundance of information about HDS online. However, they were concerned about the credibility of this information, which posed a challenge for them to conduct SL-HDS. Thus, the students identified that skills in appraising information and selecting references are vital for them to conduct SL-HDS. The perceived need to have a skill in information appraisal was more prominently noted among the first- and second-year students. 

#### 3.2.3. Social/Professional Role and Identity

The students agreed that it is part of their responsibilities as pharmacy students to conduct SL-HDS. They also admitted the need for pharmacy students to have sufficient knowledge about HDS. Some students anticipated that they would frequently encounter HDS users after graduation due to the widespread use of the products among the public. Therefore, they felt the need to equip themselves with HDS knowledge to be confident in discussing HDS with consumers. 

#### 3.2.4. Belief about Capabilities

Generally, the students were confident in conducting SL-HDS on their own. SL-HDS was perceived as an easy method to find out more about HDS. Nevertheless, the students cited the challenges in utilising the information they gathered from SL-HDS. Many felt that they needed to be guided, especially by their lecturers, to validate their findings. Furthermore, HDS was viewed as a broad topic by some of the students. They also often encountered contradicting information about the topic, which made them unsure about what they had learnt through their SL. It should be noted that the need for guidance for SL-HDS was only expressed by the first- and second-year students. 

#### 3.2.5. Beliefs about Consequences

Generally, the students saw several positive benefits of conducting SL-HDS, including knowledge gain in HDS. With this knowledge, the students stated that they would be more confident in responding to inquiries about HDS or explaining to others about the products. Additionally, several students mentioned that SL-HDS impacted positively on their attitude towards HDS. 

Despite these expressions of positive outcomes from SL-HDS, students from all study years expressed concern that SL-HDS may result in incorrect interpretations around HDS if they selected the wrong references or used unreliable sources of information. 

#### 3.2.6. Motivation and Goals

Students across the study years perceived the need to equip themselves with HDS knowledge, especially when the topic was not adequately discussed in their formal learning. This was the main motivation for the students to engage in SL-HDS. Regardless of their positive motivation to engage in SL-HDS, the packed schedule of their undergraduate programme required the students to prioritise their attention to the main topics in the programme. Hence, they found it difficult to perform SL-HDS even during their free time. 

#### 3.2.7. Memory, Attention, and Decision Processes

In general, SL-HDS was not routinely performed by the students. However, they mentioned several situations in which SL-HDS was conducted spontaneously. Several students admitted they conducted SL-HDS if they were made aware of the use of HDS among the public. The students also stated that, to some extent, they embarked on SL-HDS following the use of HDS by their close relatives and friends. 

#### 3.2.8. Environmental Context and Resources

The main constraint for students in terms of performing SL-HDS was a lack of time. As mentioned previously, the students expressed concern about their tight schedules, which made it difficult for them to allocate time for SL-HDS. On the other hand, several environmental contexts and resources were perceived as facilitators for the students to conduct SL-HDS, with the main facilitator being internet access. The students perceived the internet as an important resource for HDS information, and it was regarded as more convenient than reference books. The internet was also described as facilitating the execution of SL-HDS. On that note, the students revealed that they used various online platforms and web search engines to seek information and learn about HDS. However, many students also believed that reference books on HDS are still beneficial for them to carry out SL-HDS. Some students mentioned that these reference books are essential to validate the information found online. 

In addition, most of the third-year students and all the fourth-year students affirmed that they had conducted SL-HDS to some extent during their community pharmacy placement. This was primarily due to frequent encounters with customers using HDS. During the placement programme, it was easier to perform SL-HDS due to the availability of pharmacists on site, meaning the students had the opportunity to ask questions and clarify information about HDS. 

#### 3.2.9. Social Influences

The students were cognizant of the fact that the public expect them to be able to advise users about HDS. Therefore, they need to be knowledgeable about the products. Due to the limited content on HDS in the curriculum, the students sensed that it was necessary to conduct SL-HDS. Surprisingly, only one student mentioned that she was encouraged by lecturers to conduct SL-HDS. 

#### 3.2.10. Emotions

Some students indicated that they enjoyed performing SL-HDS, especially when they discovered new knowledge about HDS. Consequently, this prompted them to further engage in SL-HDS. Some students felt contented due to the perceived positive outcomes from SL-HDS (e.g., knowledge gained about HDS, ability to answer inquiries related to HDS).

#### 3.2.11. Behavioural Regulation

Several students argued that the inclusion of SL-HDS in their formal learning or curriculum would be important for them to engage more frequently in the activity. They recommended that the lecturers discuss issues regarding HDS to promote their interest in the topic and highlighted the need to be assigned some SL-HDS tasks to execute.

#### 3.2.12. Nature of the Behaviours

Despite some of the students having conducted SL-HDS to some extent, they still admitted that there is presently no obligation from the faculty for them to engage in SL-HDS. In general, students conducted SL-HDS during their free time and on their own initiative. Although SL-HDS was not compulsory, it was still perceived by many students as their main method to learn about HDS. This perception was based on the fact that HDS topics were not adequately covered in their curriculum.

## 4. Discussion

This exploratory qualitative study explored SL-HDS practices among a sample of pharmacy students and their beliefs on the factors associated with the activity through the lens of the TDF. The strength of this study relates to the application of the TDF, which permitted an extensive exploration of perceived facilitators of and barriers to SL-HDS from the pharmacy students’ perspectives. In this study, SL-HDS was generally self-initiated without specific instructions from the lecturers. Many students were motivated to engage in SL-HDS mainly due to the perceived need to acquire more information about HDS. The awareness of HDS use among the public, or among their family and friends, prompted them to conduct SL-HDS. As voiced by the students, an immediate positive outcome of SL-HDS was that it helped them to keep abreast with recent updates about HDS. In the same vein, the students agreed that, by performing SL-HDS, they gained knowledge about HDS in various aspects (e.g., indications, adverse effects, and HDS–drug interactions), which improved their confidence in discussing the products with others. 

The present study found that, through SL-HDS, students were made aware of their knowledge gap, subsequently prompting them to take further steps to learn more about the topic. This is favourable since pharmacists are required to familiarise themselves with HDS to be able to advise consumers on the appropriate and safe use of the products [19]. Students’ positive motivation to conduct SL-HDS, along with the reported perceived benefits, showed their readiness and willingness to engage in the activity. This finding corroborates the reports from a previous survey in which pharmacy students generally welcomed the inclusion of education relating to HDS in their curricula [39]. Students’ readiness and willingness to engage in SL-HDS also creates an opportunity to incorporate SL-HDS into formal learning. This is encouraging, since SL skills are essential to transform pharmacy students into active, independent, and life-long learners [40]. Independent and life-long learning skills, especially in the context of HDS, are beneficial for the students in the long run. More importantly, such skills are necessary during their career, particularly in the community and hospital pharmacy sectors in which HDS consumers can be frequently encountered [41].

In this study, students expressed positive attitudes towards SL-HDS. However, their degree of engagement in the activity was affected by other tasks. Due to busy learning schedules, many students in this study had to prioritise performing the other activities required in their pharmacy programme. This finding is consistent with the study by Tunney and Bell (2011), in which the concept of SL was not fully embraced by pharmacy students due to their tight schedules [42]. Therefore, to promote students’ participation in SL-HDS, pharmacy faculties should incorporate the learning method in existing teaching (e.g., during pharmacology or pharmacotherapy classes). As an example, SL-HDS can be implemented in a class on the pharmacotherapy of endocrine disorders: students can be assigned to conduct SL on the evidence-based use of HDS for patients with endocrine diseases [43]. Such a practice will assist students to integrate the knowledge of conventional medicines and HDS.

Nevertheless, it can be argued that pharmacy curricula are usually already packed, which may hinder the incorporation of SL-HDS into formal teachings. Thus, introducing SL-HDS in the form of flexible learning, such as through online learning, may be more feasible [44]. This learning mode might be favourable for the students since they appeared to be comfortable using the internet for SL-HDS and for obtaining information about HDS [45]. In this regard, online learning modules or courses (e.g., massive open online courses) related to the HDS could be developed and offered.

The FGIs revealed that SL-HDS was mainly performed without the help of others. The students usually explored the topics through online resources. However, they expressed their concerns regarding their ability to consolidate the information gathered from their SL-HDS. This highlights the need for students to be guided by lecturers to ensure they gain optimum outcomes from their SL-HDS. Therefore, it is recommended that SL-HDS be developed as a guided module. In fact, an ideal SL should not be conducted as an individualistic activity or in a way that students are isolated from others. As described by Garrison (1997), SL should include collaborative experiences in which facilitators provide support, direction, and guidance for the attainment of the necessary educational outcomes [46].

As noted in the present study, students encountered various resources on HDS and they were uncertain of their credibility, thus raising concern about making a wrong conclusion from their SL-HDS. Therefore, the faculties should introduce reliable reference resources for students to obtain current evidence-based information on HDS. Examples of these resources include the Health Care Professional Allied and Complementary Medicine Database (AMED), Micromedex AltMedDex, and the Journal of Natural Products [41]. More importantly, students need to be equipped with critical appraisal skills that allow them to assess the literature on HDS when performing SL-HDS. Of note, 40% of the students (with at least one from each student group) cited the importance of and desire to have this critical appraisal skill. Aside from reflecting a gap in the current teaching, this illustrates students’ interest in being trained in that area, so that they become more competent in critical appraisal. This suggests the need for continuous critical appraisal training during pharmacy education.

Interestingly, all students in the third and fourth years who had completed their community pharmacy placement stated that they engaged in SL-HDS during their placement. This finding provides some insights. Firstly, students may be prompted to conduct SL-HDS due to: (1) frequent inquiries regarding HDS, since most community pharmacies stock HDS; (2) the limited discussion on HDS between preceptors and students due to the hectic working environment and busy schedule; and (3) the focus of a community pharmacy placement, which is mainly on the management of pharmaceutical drugs, community pharmacy business management, and its laws and regulations [47]. Secondly, since our study finding showed that students are willing to conduct SL-HDS and acknowledged its benefits during their community pharmacy placements, there is an opportunity to introduce SL-HDS as part of the learning components in these placements. It is worth noting that SL-HDS during a community pharmacy placement is favoured by the students, since they can confirm the information they gather with community pharmacy preceptors on site.

Despite the numerous benefits of SL-HDS mentioned by the students, the willingness of the students to perform the activity, and the available opportunities to incorporate SL-HDS in current pharmacy teachings, the present study showed that there was limited attention from the faculty to promote SL-HDS. Only one of the students interviewed mentioned that the lecturers encouraged them to engage in SL-HDS. Therefore, it is pertinent to instil awareness among lecturers of the potential benefits of SL-HDS and to encourage them to incorporate SL-HDS in their teachings.

### Limitations of the Study

Although this work is novel in the topic of SL-HDS, the limitations of this study are well acknowledged. This qualitative study only included a small number of pharmacy students from one public university in Malaysia. Therefore, the findings regarding the facilitators of and barriers to conducting SL-HDS cannot be generalised to pharmacy students in other universities or in other countries. Pharmacy students in other universities, especially those in other countries, may have unique facilitators and barriers to conducting SL-HDS due to the differences in their pharmacy education structure and learning environment. Thus, this study could have been further strengthened by recruiting pharmacy students from other universities to obtain multiple and diverse perspectives regarding the topic [37]. Additionally, future studies can be conducted in other nations so that comparisons of findings between countries can be undertaken. With that being said, the findings from this study may inform other pharmacy education providers, locally or abroad, about the potential strategies to promote SL-HDS among pharmacy students at their institutions. Moreover, since the students recruited in this study were diverse in terms of their socio-demographic profiles, the transferability of the findings, especially to other local settings, is plausible.

As in other FGIs, there was a variation in the level of participation among students. For instance, while some students were more active in speaking and sharing their experiences, others were relatively passive. Some students might have been reluctant to speak if others had already mentioned similar opinions. Furthermore, although the students were offered anonymity and confidentiality, the topic of discussion that revolved around learning behaviours may subject their responses to social desirability bias. On the other hand, it is worth noting that students also mentioned circumstances in which SL-HDS was not favourable (e.g., during busy times, when having other tasks to complete), suggesting that social desirability bias was, in a way, minimised. While four FGIs were adequate to identify the important issues regarding SL-HDS, additional FGIs may be needed to fully understand these issues [48]. Furthermore, it is important to note that SL practices, and the facilitators and barriers described in this study, may only apply to HDS topics; students’ SL practices for other topics in the pharmacy curricula may be different. Lastly, the correlation between students’ beliefs about SL-HDS and their engagement in the activity could not be elucidated in this study. For that purpose, a quantitative survey using an instrument designed for a larger sample size of pharmacy students would be more appropriate.

## 5. Conclusions

This study explored the beliefs of pharmacy students about SL-HDS and identified various factors influencing their engagement in the activity. Overall, the students acknowledged the benefits and were willing to conduct SL-HDS. The participants perceived that their engagement in SL-HDS is compatible with the role of pharmacy students. However, they expressed limited confidence in assessing literature or information about HDS, thus emphasizing the importance of equipping students with critical appraisal skills. This study also uncovers opportunities to continuously incorporate SL-HDS elements in pharmacy education. The findings from this study might serve as input to inform several strategies around promoting SL-HDS among pharmacy students. This may include the incorporation of SL-HDS elements into community pharmacy placement and providing training to enhance students’ critical appraisal skills. Additionally, lecturers could be trained to assist the implementation of effective SL-HDS. 

## Figures and Tables

**Table 1:** Discussion questions for the focus group interviews and the corresponding theoretical domains.

Domain	Domain Definition	Interview Questions
**Knowledge**	An awareness of the existence of something.	• In your opinion what is SL-HDS? • Why do you think SL-HDS should be done? • How should SL-HDS be done?
**Skill**	An ability or proficiency acquired through practice.	• What are the skills required for you to conduct SL-HDS? • Do you feel that you have the skills required to conduct SL-HDS? • What additional skills or training might you need to conduct SL-HDS?
**Social/Professional Role and Identity**	A coherent set of behaviours and displayed personal qualities of an individual in a social or work setting.	• As a pharmacy student, tell me about how you feel about conducting SL as a means of improving your knowledge about HDS. • Is SL-HDS fits your role as a pharmacy student? • Is conducting SL-HDS something that a pharmacy student should be expected to do?
**Beliefs about Capabilities**	Acceptance of the truth, reality, or validity about an ability, talent or facility that a person can put to constructive use.	• How easy or difficult is it to conduct SL-HDS? • Is there anything that helps you, or makes it easier for you to conduct SL-HDS? • What problems/difficulties do you encounter when conducting SL-HDS? • What might help you to overcome these problems/difficulties? • How confident are you about conducting SL-HDS, despite these difficulties?
**Beliefs about Consequences**	Acceptance of the truth, reality or validity about outcomes of a behaviour in a given situation.	• What do you think are the benefits of SL-HDS? • What do you think are the disadvantages of conducting SL-HDS? • Do the advantages of conducting SL-HDS outweigh the disadvantages?
**Motivation and Goals**	The relative priority given to one issue compared to other demands; mental representations of outcome or end states that an individual wants to achieve.	• How much do you want to conduct SL-HDS? • How much do you feel that you need to conduct SL-HDS? • Would by conducting SL-HDS prevent you from or make it difficult for you to achieve any of your other responsibilities as a pharmacy student (e.g., studying other subjects, doing other activities, etc.)?
**Memory, Attention, and Decision Processes**	The ability to retain information, focus selectively on aspects of the environment and choose between two or more alternatives.	• Is SL-HDS your main method of improving your knowledge about HDS? • Is SL-HDS something that you spontaneously do? • Does conducting SL-HDS ever slip your mind?
**Environmental Context and Resources**	Any circumstance of a person’s situation or environment that discourages or encourages the development of skills and abilities, independence, social competence, and adaptive behaviour.	• What are the main barriers and facilitators for you to conduct SL-HDS? • Which resources help you to conduct SL-HDS? • Lack of which resources makes it difficult for you to conduct SL-HDS? • Are there any competing tasks or time constraints that might influence whether you conduct SL-HDS or not?
**Social Influences**	Those interpersonal processes that can cause individuals to change their thoughts, feelings, or behaviour.	• Are there any individuals, groups, or organisations that approve you to conduct SL-HDS? • In what ways do the views of others affect your conduct of SL-HDS?
**Emotion**	A complex reaction pattern, involving experimental, behavioural, and physiological elements, by which the individual attempts to deal with a personally significant matter or event.	• What do you feel when you conduct SL-HDS? • Do your feelings when you conduct SL-HDS influence whether or how you conduct SL-HDS? If so, how?
**Behavioural Regulation**	Anything aimed at managing or changing objectively observed or measured actions.	• What do you need to do to conduct SL-HDS? • Are there procedures, systems or methods that might encourage you to conduct SL-HDS?
**Nature of the Behaviour**	Some new behaviours are very similar to current behaviour and are easier to implement than new behaviours that require a dramatic change in life.	• Please describe what it means by conducting SL-HDS to you. • Is SL-HDS an expected part of your learning?

SL, self-learning; HDS, herbal and dietary supplements; SL-HDS, self-learning about herbal and dietary supplements.

**Table 2:** Characteristics of students.

Characteristics	*n* (%)
Average age (±standard deviation)	22.6 (±1.7)
Gender	
Male	4 (20)
Female	16 (80)
CGPA category	
<3.00	5 (25)
3.00–3.49	7 (35)
3.50–4.00	8 (40)
Currently using HDS	
Yes	6 (30)
No	14 (70)
Enrolled in TM elective course	
Yes	7 (35)
No	8 (40)
Non-applicable ^a^	5 (25)

CGPA, cumulative grade point average; HDS, herbal and dietary supplements; TM, traditional medicine; ^a^ TM course only offered in the second semester of the second year of study.

**Table 3:** Frequency of specific beliefs regarding SL-HDS according to the Theoretical Domains Framework.

Specific Belief by Domain	Total Frequency of Mentions	Sample Quote(s)
**1. Knowledge**		
a. SL-HDS is a method to gain knowledge about HDS.	10	“For me, self-learning about herbal and dietary supplements is when I took my time to learn about it with no one is directly teaching.”—PS09, female, second-year student.
b. SL-HDS is informal learning about HDS.	10	“We look it up on the internet and try to understand it.”—PS14, male, third-year student.
c. SL-HDS is learning without supervision.	7	“It’s a learning without supervision.”—PS11, female, third-year student.
**2. Skills**		
a. Students require an adequate knowledge base in pharmacy-related topics to conduct SL-HDS.	16	“We need to be knowledgeable in pharmacology so that we can understand the action of the products in our bodies.”—PS15, female, third-year student.
b. Students require critical appraisal skills to conduct SL-HDS.	7	“We are doing it on our own, so we need to know how to choose our references, not using any sources like the blogs.”—PS01, female, first-year student. “The challenge is to make sure the sources are reliable.”—PS05, female, first-year student.
**3. Social/professional role and identity**		
a. SL-HDS is compatible with the role of pharmacy students.	15	“We are future pharmacists, we don’t only provide advice on the use of modern medicines. We should be able to discuss herbal and dietary supplements too, so we need to be knowledgeable in both.”—PS07, female, second-year student.
**4. Beliefs about capabilities**		
a. Students are confident in conducting SL-HDS.	12	“I am confident I can do it.”—PS 14, male, third-year student.
b. SL-HDS is perceived as an easy mode of learning.	12	“Even at the bus stop we can do it. If I want to know about it, I will just google.”—PS03, male, first-year student.
c. Students are not confident in consolidating information gathered from SL-HDS.	10	“In my opinion, there is no problem with learning myself. However, we usually get the information from the internet, so we have to confirm what we found with someone who knows about it. Also, the topic is broad. Sometimes we are not sure what to cover. Perhaps, the lecturers can guide us.”—PS05, female, first-year student. “There are things that we don’t understand and not clear about. We can learn it on our own but there must be someone who can guide us.”—PS03, male, first-year student.
**5. Beliefs about consequences**		
a. Students gain new knowledge about HDS.	13	“With the knowledge gained, we can educate others about herbal and dietary supplements.”—PS12, female, third-year student.
b. Students gain confidence in answering inquiries about HDS.	4	“When we have the knowledge, we have the confidence to talk about it.”—PS09, female, second-year student.
c. SL-HDS promotes a positive attitude towards HDS.	5	“You know, we would realise that some herbal and dietary supplements may have health benefits.”—PS05, female, first-year student.
d. SL-HDS results in the wrong interpretation about HDS.	15	“Maybe we will get the wrong information or choose a wrong information source. Maybe we misinterpret the information.”—PS01, female, first-year student.
**6. Motivation and goals**		
a. Students conduct SL-HDS due to the perceived need to gain/update knowledge about HDS.	16	“I do it to increase my knowledge about herbal and dietary supplements.”—PS19, female, fourth-year student. “I need the knowledge, but I can’t just rely on classes, so I have to do it.”—PS04, male, first-year student.
b. SL-HDS competes with students’ time.	15	“It’s quite hard to find the time to do it, there’s still a lot of studying need to be done on pharmaceutical drugs.”—PS15, female, third-year student.
**7. Memory, attention, and decision processes**		
a. Awareness of the common use of HDS by the public encourages students to conduct SL-HDS.	12	“I know that there are a lot of people out there who are using herbals. These products are everywhere. That means herbals are highly sought after, and many believe in the products. It triggers me to do some research about it.”—PS14, male, third-year student. “Some products are highly used by the public, it makes me want to know about the products.”—PS10, female, second-year student.
b. HDS use by family and friends triggers students to conduct SL-HDS.	11	“My father uses some dietary supplements. It makes me curious about these products, so I conduct some research to know about it.”—PS15, female, third-year student. “I have friends who use some herbal products that they found on the internet. It makes me think. Is it okay to consume it? Like those skin whitening supplements. I have to look it up.”—PS04, male, first-year student.
**8. Environmental context and resources**		
a. Time is a factor in students’ decision to conduct SL-HDS.	11	“The challenge is to find the time.”—PS08, female, second-year student. “We need to do a lot of readings, so it takes so much of our time.”—PS07, female, second-year student.
b. Access to the internet makes SL-HDS easier.	19	“Mostly we use the internet, it’s easier. If we go to the library, the books are thick, not appealing.”—PS14, male, third-year student.
c. Reference books assist students understanding about HDS during SL-HDS.	11	“Usually, we google it up first, but we need to refer to books to make sure. There’s one good book on herbals at the library.”—PS10, female, second-year student.
d. The community pharmacy attachment encourages students to conduct SL-HDS.	8	“We got many customers buying the products, so we have to look it up. What are the benefits? Why do they consume it?”—PS19, female, fourth-year student. “During the community pharmacy attachment, we can ask the pharmacists.”—PS12, female, third-year student.
**9. Social influences**		
a. Expectations from the public for pharmacy students to know about HDS promotes SL-HDS.	9	“People would expect us to know about herbal and dietary supplements, so we need to have the knowledge. In this program, we only have an elective course for it, so we have to learn it by ourselves.”—PS15, female, third-year student.
b. Lecturers.	1	“The lecturers would expect us to learn about herbal and dietary supplements.”—PS15, female, third-year student.
**10. Emotion**		
a. Students enjoy conducting SL-HDS.	7	“I enjoyed doing it. Like me, previously, I don’t even know that Gingko biloba can interact with warfarin, but when I did some research, it does!”—PS13, female, third-year student.
b. Students feel satisfied after conducting SL-HDS.	5	“It gives me satisfaction when I know about it, when I can explain to others.”—PS05, female, first-year student.
**11. Behavioural regulation**		
a. Incorporation of SL-HDS into formal learning may allow more frequent conduct of the activity.	9	“I think the lecturers can discuss some issues about the products. Recently, there is a product that was withdrawn from the market because it contains steroids. They can discuss this in class, it’s interesting.”—PS11, female, third-year student. “If learning about herbals can be assigned to us that would make us do it more often.”—PS16, female, fourth-year student.
**12. Nature of the behaviour**		
a. There is no obligation to conduct SL-HDS.	11	“I’ll do it when I want to. No specific time. Up to me.”—PS10, female, second-year student.
b. SL-HDS is a common learning method to learn about HDS.	12	“It’s not usual for us to discuss herbal and dietary supplements in class, so we need to learn by ourselves.”—PS16, female, fourth-year student. “Sometimes I don’t understand what I learn (about HDS) from class, so I have to do some self-studying.”—PS02, female, first-year student.

HDS, herbal and dietary supplements; PS, pharmacy student; SL-HDS, self-learning about herbal and dietary supplements.

## Data Availability

The data presented in this study are available on request from the corresponding author. The data are not publicly available due to privacy.

## Abbreviations

**HDS:** Herbal and dietary supplements; **CAM:** Complementary and alternative medicine; **HS:** Herbal supplements; **DS:** Dietary supplements; **US:** United States; **SL-HDS:** Self-learning about HDS; **SL:** Self-learning; **TDF:** Theoretical Domain Framework; **FGI:** Focus group interview; **CGPA:** Cumulative grade point average; **AMED:** Allied and Complementary Medicine Database.
